# Development of Osmotically Controlled Mucoadhesive Cup-Core (OCMC) Tablet for The Anti-Inflammatory Activity

**Published:** 2010

**Authors:** Hitesh Ranchhodbhai Patel, Madhabhai Manordas Patel

**Affiliations:** *Department of Pharmaceutical Technology, S. K. Patel College of Pharmaceutical Education and Research, Ganpat University, Gujarat, India.*

**Keywords:** Osmotic cup-core tablet, Aceclofenac, Mucoadhesion, Drug release, Anti- inflammatory activity

## Abstract

The aim of the present study was to prepare and evaluate an osmotically controlled mucoadhesive cup-core (OCMC) containing aceclofenac. A special technique was used while preparing an OCMC. Stability of OCMC was determined in natural human saliva, and it was found that both pH and device are stable in human saliva. OCMC was evaluated by weight uniformity, thickness, hardness, friability, swelling, mucoadhesive strength and in vitro drug release. Swelling index was higher with formulations containing hydroxypropyl methylcellulose (HPMC) K4M alone, and it decreases with its decreasing concentration in the OCMC. The in vitro drug release studies showed a release with the composition of formulation up to 12 h. The mechanism of drug release was found to be zero order kinetics with diffusion controlled drug release. It has shown significant anti-inflammatory activity (P<0.001) and no hypersensitive reaction. It can be concluded that by changing the content of OCMC system, a desire effect is generated and it overcomes the drawback associated with the conventional buccal adhesive tablet.

## Introduction

The oral mucosa can be categorized into sublingual, gingival, and buccal mucosa through which oral transmucosal delivery can be achieved. Absorption of therapeutic agents from the oral cavity provides a direct entry of such agents into the systemic circulation, thereby avoiding the first-pass hepatic metabolism and gastrointestinal degradation. However, the buccal route of drug delivery has received much more attention because of its unique advantages over other oral transmucosal routes (1). Aceclofenac is used in the management of many inflammatory conditions (2) but because of its short biological half-life and hazards of adverse gastrointestinal (GI) reactions (3), the development of oral mucoadhesive sustained-release formulations of this drug is desirable in order to achieve improved therapeutic efficacy and patient compliance (3). Various buccal adhesive dosage forms, such as discs, microspheres, and bilayered tablets, have been thoroughly prepared and reported by several research groups. 

However, limited studies exist on novel devices that are superior to those of conventional buccal adhesive systems for the delivery of therapeutic agents through buccal mucosa. The purpose of this work was to develop a controlled porosity osmotic pump system for delivery of aceclofenac. The system was composed of a cup-core tabletand a rate-controlling coating membrane. This study mainly focused on the preparation of the dosage forms using a special technique to deliver the drug via oral cavity. Aceclofenac was chosen as a model drug candidate for their suitability of this route with all aspect. In vivo release studies were also performed to correlate results.

## Experimental

**Materials**

Aceclofenac was obtained as gift sample from Torrent Pharmaceutical, Gujarat; HPMC K4M, carbopol 934P, PVP K30 were purchased from Sigma Chemicals, Mumbai. All the chemicals and reagents were of analytical grade.

*Preparation of OCMC tablet*

Composition of the cup-core tablet is shown in Table 1. Polyvidone K30 was diluted to 20% (w/v) with ethanol. Aceclofenac and osmotic agents were blended evenly and sieved through 80-mesh sieve (180 mm). Polyvidone K30 solution was added gradually and mixed uniformly with the mixture. The wet mess was dried at 50-60°C for 4 h. The dried mess was powdered with a mortar and passed through 80-mesh sieve. The obtained powder was lubricated with magnesium stearate. The core tablet was compressed into 200 mg tablets (Cadmach single station tablet machine, 8-mm round, flat punches). To prepare the cup, volume of the die cavity (12-mm round, flat punches) was adjusted equivalent to the weight of 350 mg; then, the inner layer was placed manually at the central of bottom layer; eventually, the remaining volume of the die cavity was filled with 350 mg outer layer powder and compressed with a maximum compressing force of the tablet machine to obtain the cup-core tablet (4). 

**Table 1:** Composision of the cup-core tablet

**Ingredients**	**Inner layer (%, w/w)**				**Outer layer** **(%, w/w)**
	**F1**	**F2**	**F3**	**F4 **	
**Aceclofenac**	25.0	25.0	25.0	25.0	-
**Sodium chloride**	69.0	59.0	-	-	-
**Sodium phosphate**	-	-	69.0	59.0	-
**Polyvidone K30**	5.0	5.0	5.0	5.0	-
**Magnesium stearate**	1.0	1.0	1.0	1.0	3.0
**Lactose**	-	10.0	-	10.0	
**HPMC K4M**	-	-	-	-	50
**Carbopol 934P**	-	-	-	-	50

*Coating*

 Different coating solutions were prepared. PEG 400 was selected as the optimal pore forming agent concentration; other coating solutions were used to study the effects of amount and type of pore forming agents on drug release. Average weight gain after coating was controlled at different amounts to study the influence of coating thickness on drug release. The outer later of core tablet was coated with cellulose acetate (CA) and PEG 400 as the optimal pore forming agent by spray coating at one side with 8.5 cm in diameter (5). 

*In vitro drug release*

To study the effect of pH on release profile, release studies were conducted in dissolution medium with different pHs (simulated gastric fluid, pH 1.2; phosphate buffer, pH 6.8; deionized water, pH 7.0; phosphate buffer, pH 7.4) at a rotation speed of 100 rpm. The dissolution profile at different pHs and bio-adhesion property of OCMC tablet were determined. Release studies were carried out in a defined medium during the entire study. All experiments were repeated three times. Samples of 5 mL were withdrawn at specified time points (0, 1, 2, 4, 6, 8, 10, 12 h) and replaced with fresh dissolution medium. Obtained samples were properly diluted and analyzed by UV-absorption measurement. The in vivo studies were conducted and student *t *test (paired and one-sided) was performed to determine the level of significance (6).

*Swelling studies*

OCMCs were weighed individually (designated as W_1_) and placed separately in petridishes containing 4 mL of phosphate buffer solution (pH 6.6). At regular intervals (0.5, 1, 2, 3, 4, 5, 6 h), the NBASs were removed from the petridishes and excess surface water was removed carefully using the filter paper. The swollen NBASs were then reweighed (W_2_), and swelling index (SI) was calculated using the following formula (7).

$\mathrm{SI}=\frac{\left(W2-W_{1}\right)}{W_{1}}$

*Stability of OCMCs*

Stability studies of OCMC were performed in normal human saliva using the optimized formulation (F2) selected based on the results of swelling, release, and mucoadhesion strength studies. Briefly, the human saliva was collected from humans (aged 18-55) and filtered. OCMCs were placed in separate petridishes containing about 5 mL of human saliva and placed in a temperature-controlled oven for 6 h at 37±0.2°C. At regular time intervals (0, 1, 2, 3, 6 h), it was examined for change in color and shape, collapse of the OCMC, and drug content. The experiments were repeated in triplicate (n=3) in a similar manner (8).

*In vitro bio-adhesion test*

A double pan physical balance was taken and both the pans were removed. The left pan was replaced with a brass wire. The right pan was replaced with a lighter pan. In the left pan polypropylene block was placed. The goat cheek pouch was carefully excised without removing connective and adipose tissue and stored in saline solution. The left side pan was placed in the beaker contained phosphate buffer of pH 6.6 and kept at 37 ± 1°C. The film was taken and attached to upper polypropylene cylinder, and goat cheek pouch was attached on the lower polypropylene block. A preload weight of 30 g was placed on the left pan of the balance for 10 min. The weights were then removed slowly and weights were added slowly in increasing order to the right pan till the patch separates from the mucosal surface. The weights required for complete detachment of the film from mucosal surface was noted. Average of three determinations was calculated and applied standard deviation (9, 10).

**Figure 1:**
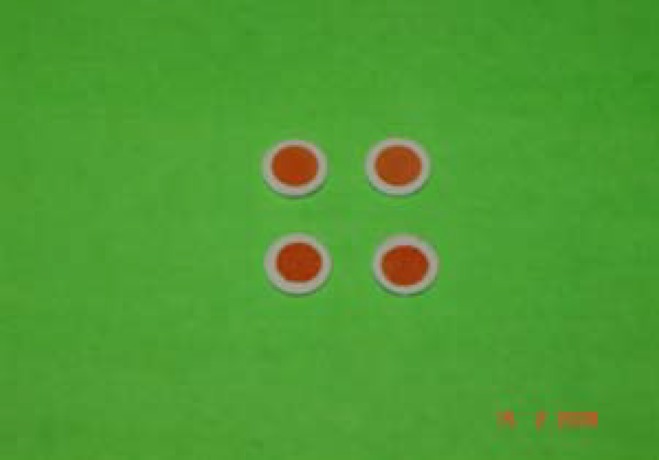
Photograph of OCMC tablets

*In vivo studies*

*Anti-inflammatory activity*

This experiment was carried based on the method described elsewhere (11-13). Twelve albino rats of either sex having weight between 170 and 200 g were divided into two groups of six animals each. The ventral surface of the animal was depilated; one group was treated as control, and the other group was treated as test. The F2 containing the dose of aceclofenac calculated on the basis of surface area of the animal under study was stuck on the animal; then, a backing laminate of aluminium foil was placed over the tablet, and it was kept intact with the help of adhesive tape. A 2% v/v formalin solution was used as chronic inflammogen to induce inflammation. A mark was made on hind paw just behind tibiotarsal junction, so that every time the paw was dipped in the mercury column up to the fixed mark to ensure constant paw volume. After 1 h, the inflammogen was injected subcutaneously into the paw of all animals. The paw volumes of all the animals, both control and test groups were measured by using plethysmograph at selected interval of time and the results were tabulated. From the above results, the percentage reduction in oedema volume was calculated. Finally, the activity of the formulation was statistically analyzed by student *t *test. 

**Table 2:** Kinetic values of drug release from OCMC systems

**Formulation **	**Zero order equation**	
	**n**	**r**
F1	2.225	0.9971
F2	1.521	0.9975
F3	3.661	0.9963
F4	3.395	0.9977

## Result and Discussion

Average hardness of the cup-cores was found to be 13±0.5 kg/cm^2^ (Pfizer hardness tester). Drug content of the cup-cores was within the limits of 95%-105%. OCMC did not exhibit change in color or shape, suggesting the satisfactory stability of the drug and device in the human saliva; it also did not rupture in human saliva at the end of experiment. Maximum swelling was attained at the 5^th^ h, after which polymer started eroding slowly in the swelling medium. High amount of water uptake by HPMC K4M at a faster rate might have resulted in higher rate and extent of swelling. The bioadhesion strength of 8.2-9.0 was obtained with all formulations, and it was considered to be sufficient for buccal formulation. 

**Figure:**
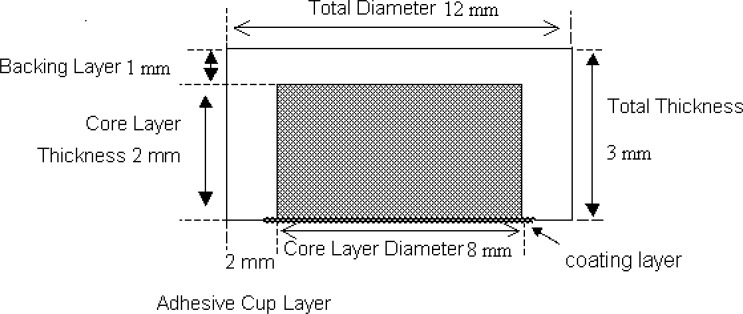


**Figure 3:**
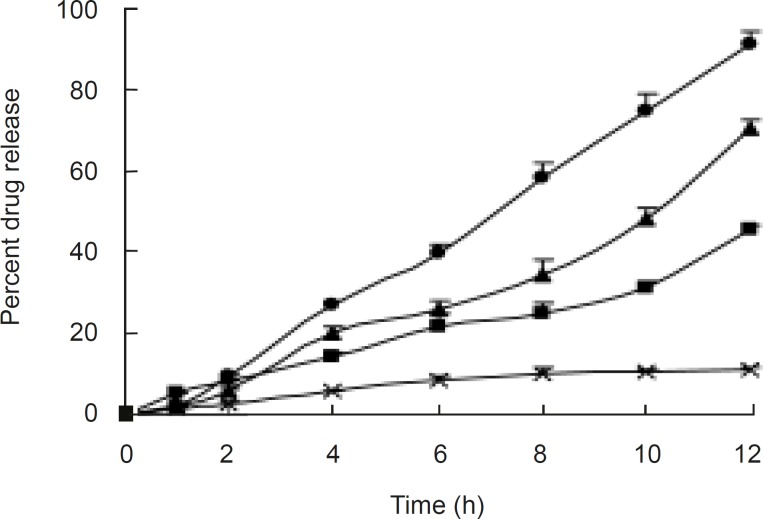
Release profiles of formulations with different amount of CA and PEG 400 Composition of coating membrane: ×, CA: PEG 400 (72.7%:18.2%); ■, CA:PEG 400 (63.6%:27.3%); ▲, CA:PEG 400 (54.5%:36.4%); ●, CA:PEG400 (45.5%:45.5%).

It was observed from release profiles (Figure 3) that an increase in membrane thickness affected the drug release rate inversely. All the tablets exhibited constant and controlled drug release profiles from first hour onwards. Owing to the presence of porosigenic agents such as PEG 400 and CA, aceclofenac release from osmotic matrix tablets showed marginally increased and controlled release from OCMC tablet, and it followed the zero order kinetics with correlation coefficients between 0.9829 and 0.9977;moreover, mechanism of drug release was diffusion controlled. By changing the concentration of CA the rate of release was changed.

**Figure 4:**
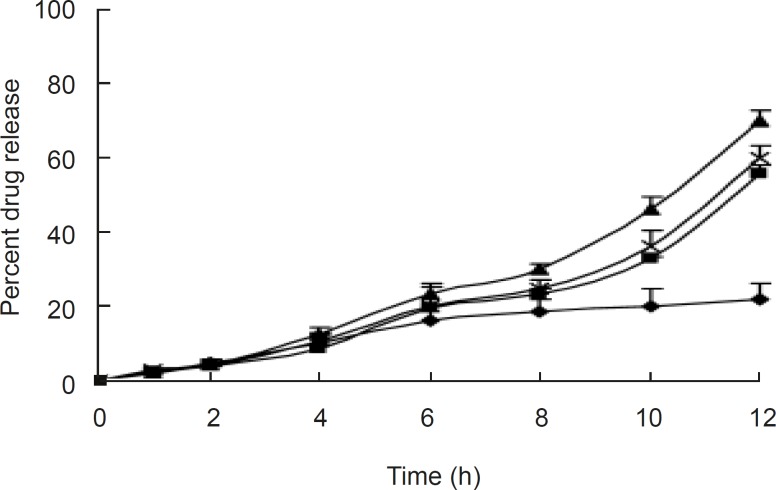
Effect of pH on release from the optimized formulations pH of the dissolution medium: ♦, 1.2; ■, 6.8; ▲, 7.0; ×, 7.4

The release profiles of aceclofenac in the different dissolution medium indicated no difference, as shown in Figure 4. The surface pH values were found to be between 6.2 and 6.8 in all the formulations, indicating that the formulations were compatible with oral mucosa. The anti-inflammatory activity and hypersensitivity reaction of the selected tablet (F2) were determined; it showed 46.14 % reduction in oedema volume at the end of 12 h with mean value of 0.817, which indicates significant anti-inflammatory activity compared to the control (P <0.001). The selected OCMC system showed no sign of allergic or hypersensitivity reactions for seven days (Table 3). 

**Table 3:** Hypersensitivity reactions

**Time (day)**	**F2 formulation**		
	**F**	**P**	**E**	
1	– ve	– ve	– ve	
2	– ve	– ve	– ve	
3	– ve	– ve	– ve	
4	– ve	– ve	– ve	
5	– ve	– ve	– ve	
6	– ve	– ve	– ve	
7	– ve	– ve	– ve	

– ve: no allergic manifestation observed; F: flushing of skin (redness); P: papules and wheals; E: erythema and oedema.

## Conclusion

An OCMC aceclofenac delivery system was obtained by developing this novel approach for the mucoadhesive drug delivery system. It has overcome the drawback associated with drug and shows a sustained release drug delivery. This design is superior and novel to those of conventional dosage forms in tablet or lozenge formulations, which only provide a short duration of continuous drug administration. These studies suggest that the OCMC is a suitable device for delivering various therapeutic agents through buccal mucosa.

**Figure 5:**
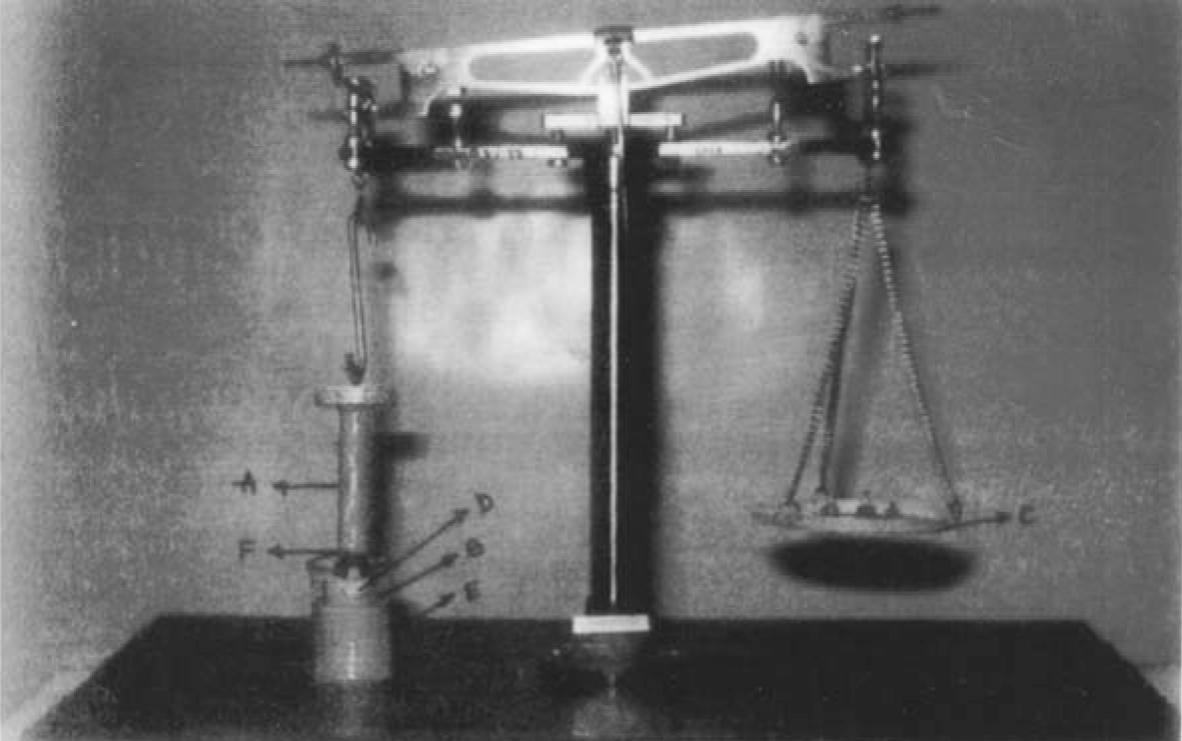
Bioadhesion equipment (Fabricated).
